# Dispersion of waves and transmission–reflection in blood vessels with structured stents

**DOI:** 10.1098/rspa.2018.0816

**Published:** 2019-03-20

**Authors:** S. Frecentese, T. K. Papathanasiou, A. B. Movchan, N. V. Movchan

**Affiliations:** 1 Department of Mathematical Sciences, University of Liverpool, Liverpool L69 3BX, UK; 2 Department of Civil and Environmental Engineering, Brunel University London, Uxbridge UB8 3PH, UK

**Keywords:** wave propagation, asymptotic analysis, periodic structures, stented arteries

## Abstract

A new model is proposed for elastic waves induced by a pulsating flow in a stenotic artery containing several stents. Dispersion properties of the waves depend on the stent structure—this feature is addressed in the present paper. Several vascular stenting procedures include overlapping stents; this configuration is also included in the model. The dispersion and transmission properties are analysed; the analytical derivations are accompanied by illustrative numerical examples.

## 1. Introduction

Biomedical acoustics is a well-developed research area encompassing the investigation of acoustic and ultrasonic wave interaction with biological systems of soft tissue, bone and organs. There are numerous established concepts and ideas used in medical diagnosis techniques and for a range of technical applications. One specific topic of interest is acoustic wave propagation in biological materials such as arterial tissue. As is often the case for the analysis of acoustic waveguides, mathematical modelling is very important in biomech- anics problems that include pulsating flow. Analytical models and numerical simulations for pulsating blood flow for an unstented artery can be found, for example, in [1–9]. In particular, it is known that the junctions between blood vessels act as scatterers, and reflection of acoustic waves from branched blood vessels is observed in routine measurements [10,11]. Accordingly reflection of acoustic waves is expected whenever the properties of the arterial wall are altered. The Bloch–Floquet waves technique has been recently employed in many applications in order to understand the dynamic properties of periodic systems [12–16]. In particular, it has been shown that there is a link between the dispersion properties of an infinite periodic system and the transmission problem for the corresponding finite system [17–19].

The approach introduced in the recent paper [20] offers a new model and theoretical concept, which considers blood vessels containing stents (alterations of the arterial properties and geometry). In addition to acoustic waves, the dynamic response of elastic solids was also considered, and thus the model was extended to include elastic waves propagating in the blood vessels reinforced by stents.

We note that the wall of a healthy blood vessel is a highly adaptable nonlinear system, and if one tries to introduce a universal mathematical approach, then the system would be described by nonlinear partial differential equations whose coefficients depend on both spatial and temporal variables. An unhealthy (clogged) or stented blood vessel loses its flexibility and linearized approaches can be adopted.

In the present paper, we focus on combining two approaches: the first one uses the Bloch–Floquet framework applied to an infinite periodic system, while the second method is based on the transmission analysis of a finite-thickness structured interface. In particular, we explore homogenization approximations, where a stented region is described by differential equations with variable coefficients.

Our approach is new in the dynamic response analysis of blood vessels, and it uncovers important phenomena attributed to transitional regimes where pulsating flow changes rapidly and hence high-order harmonics occur. The analytical nature of our approach enables us to use it effectively in conjunction with transient simulations to identify the values of parameters leading to the transitional regimes, which may be linked to vascular blockages in multi-scale stented systems. For convenience of the reader, we have also included an appendix A, which outlines the numerical method and the algorithm of simulations discussed in the paper. Also, we refer to the book [21] as the further reading material on the finite-element method.

The structure of the paper is as follows. Section 2 presents the one-dimensional model for a stented artery. Section 3 includes the calibration procedure for the one-dimensional model versus the three-dimensional blood vessel. Here are also presented the dispersion properties of the waves in stented systems for the one-dimensional model. The transmission problems for sparse stents are analysed in §4, with the comparison being made to the dispersion properties discussed in §3. The proposed model is employed in §5 extending the analysis to the case of two overlapping stents in an artery. Conclusions, together with the discussion of the analytical results and illustrative numerical simulations, are presented in §6.

## 2. A one-dimensional model for waves in a stented artery

A one-dimensional approach to model wave propagation through a stented artery was developed in [1]. The paper [1] presented an analytical model that takes into account the fluid–solid interaction in the framework of a transmission problem for a pulsating flow through a stented blood vessel. In the current paper, we present the new study which includes (i) calibration against the three-dimensional results of [20], (ii) the modelling of stents with sparse structure and (iii) the Bloch–Floquet analysis of waves in stented vascular systems.

### (a) Governing equations for blood flow in arteries

Referring to the study presented in [1], a low Mach number is assumed and the elastic displacements of the wall of the blood vessel are considered to be small. By referring to the cross-sectional averages, we consider the pressure *p*(*x*, *t*), and the velocity *u*(*x*, *t*) to be functions of the longitudinal variable *x* and the time *t*.

In our approach, we refer to a flow with a relatively low speed and hence consider a fluid, which is acting as an acoustic medium and interacting with an elastic cylindrical shell. This fully complies with the dynamic response of a blood vessel reinforced by a stent. The linearized one-dimensional wave propagation model incorporates the mass and momentum conservation equations, as follows:

2.1a $$\frac{\partial p}{\partial t}+c^{2}\frac{\rho }{A}\frac{\partial q}{\partial x}=0$$

and

2.1b $$\frac{\partial q}{\partial t}+\frac{A}{\rho }\frac{\partial p}{\partial x}=0.$$

Here *x* is the axial coordinate along the vessel, *t* is the time, *A* is the cross-sectional area, *q* = *Au* is the volumetric flow, *c* is the speed of propagation of the pulse wave, and *ρ* is the fluid density, approximately constant for a nearly incompressible fluid.

System (2.1a,*b*) is reduced to the wave equation

2.2 $$\frac{\partial^{2}q}{\partial t^{2}}-\frac{\partial }{\partial x}\left(c^{2}\frac{\partial q}{\partial x}\right)=0,$$

where *c* is constant in the homogeneous unstented artery, and *c* = *c*(*x*) is variable in the stented region. In particular, for a cylindrical homogeneous artery, the value *c*_0_ of the wave speed can be approximated (e.g. [22,23]) as

2.3 $$c_{0}^{2}\approx \frac{Eh}{\psi 2R\rho },$$

where *E* is Young's modulus of the artery, *h* is the thickness of the arterial wall, *R* is the internal radius of the artery and *ψ* is a constant depending on the artery constraint.

### (b) Model for an artery with a stent

For the stented region, alternative material properties must be considered. An additional wall stiffness due to the stent and the plaque is included in the model. Referring to (2.3), *E*, *h* and *A* are subject to variation. Assuming a reference wave speed *c*_0_ for the healthy (unstented) artery, the wave speed *c*(*x*) in the stented region is taken to be

2.4 $$c(x)=c_{0}+c_{A}+c_{B}f(x),$$

as proposed in [1]. Here *c*_*A*_, *c*_*B*_ are constants, and *f*(*x*) is a periodic function of period *L*:

2.5 f(x+L)=f(x),0≤f(x)≤1.

The constant *c*_*A*_ is the minimum variation from the healthy region value due to the stent and/or plaque, and *c*_*B*_ is associated with the maximum deviation, measured from the state *c*_0_ + *c*_*A*_, which occurs in the elementary cell.

**Figure 1.**
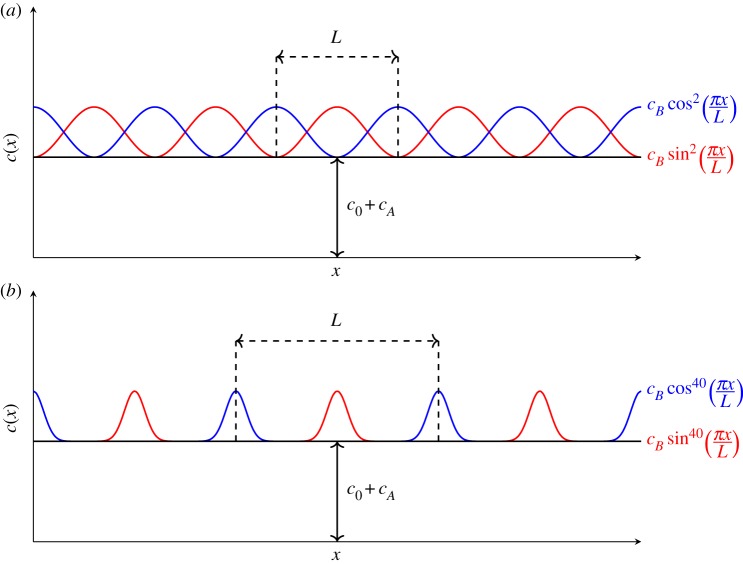
Examples of wave speed profiles for a stented artery where the exponent *s* in (2.6) and (2.7) assume different values: (*a*) *s* = 1, (*b*) *s* = 20. (Online version in colour.)

Following the model proposed in [1], *c*(*x*) is assumed to be either (figure 1)

2.6 $$c(x)=c_{0}+c_{A}+c_{B}\sin^{2s}\left(\frac{\pi x}{L}\right)$$

or

2.7 $$c(x)=c_{0}+c_{A}+c_{B}\cos^{2s}\left(\frac{\pi x}{L}\right).$$

The exponent s∈N characterizes the sparse structure of the stent.

The non-dimensional variables are introduced as follows:

2.8 $$\xi =\frac{x}{L},\;\eta =\frac{c_{0}t}{L}\;\mathrm{and}\;Q=\frac{q}{q_{0}},$$

where *L* is the length of the periodic cell and *q*_0_ is the reference value of the volumetric flow. Equation (2.2) becomes

2.9 $$\frac{\partial^{2}Q}{\partial \eta^{2}}-\frac{\partial }{\partial \xi }\left({\left(\frac{c}{c_{0}}\right)}^{2}\frac{\partial Q}{\partial \xi }\right)=0.$$

Using equations (2.6) and (2.7), in the stented area we have that

2.10 $$\frac{c(\xi )}{c_{0}}=\left\{ \begin{array}{ll} 1, & \xi \in (-\infty ,0)\\ 1+A^{2}+B^{2}\sin^{2s}(\pi \xi )\;\mathrm{or}\;1+A^{2}+B^{2}\cos^{2s}(\pi \xi ), & \xi \in (0,n)\\ 1, & \xi \in (n,+\infty ), \end{array}\right.$$

where *A*^2^ = *c*_*A*_/*c*_0_, *B*^2^ = *c*_*B*_/*c*_0_, and *n* is the number of periodic cells constituting the stent.

The solution of equation (2.9) is sought in the form *Q* = *y*(*ξ*) e^−i*ωη*^, where the function *y*(*ξ*) and the angular frequency *ω* satisfy the equation

2.11 $$\frac{d}{d\xi }\left({\left[1+A^{2}+B^{2}f(\xi )\right]}^{2}\frac{dy}{d\xi }\right)+\omega^{2}y=0.$$

Introducing *Y* such that

2.12 $$Y={\left[1+A^{2}+B^{2}f(\xi )\right]}^{2}\frac{dy}{d\xi },$$

equation (2.11) can be rearranged as follows:

2.13 $$\frac{d^{2}Y}{d\xi^{2}}+{\left[\frac{\omega }{1+A^{2}+B^{2}f(\xi )}\right]}^{2}Y=0.$$

For the stented region, the mean non-dimensional speed *C* in the unit cell can be evaluated as

2.14 $$C=\int_{0}^{1}\frac{c(\xi )}{c_{0}}\;d\xi =\int_{0}^{1}\left(1+A^{2}+B^{2}f(\xi )\right)\;d\xi =1+A^{2}+\frac{\Gamma \left(\frac{1}{2}+s\right)}{\sqrt{\pi }\Gamma \left(1+s\right)}B^{2},$$

for both choices *f*(*ξ*) = sin^2*s*^(*πξ*) or *f*(*ξ*) = cos^2*s*^(*πξ*).

## 3. Dispersion curves and stop-bands

In this section, the dispersion properties of the Bloch–Floquet waves propagating along the walls of the stented blood vessel are presented. Equation (2.11) is solved numerically using the Galerkin method with finite-element discretization together with the following Bloch–Floquet conditions applied at the ends of the unit cell *ξ* = 0 and *ξ* = 1:

3.1a y(1)=y(0)exp⁡(iK)

and

3.1b $${\frac{d^{j}y}{d\xi^{j}}|}_{\xi =1}={\frac{d^{j}y}{d\xi^{j}}|}_{\xi =0}\exp\left(iK\right),$$

where *K* is the one-dimensional Bloch–Floquet parameter (or the lattice wavenumber), and only *j* = 1 is required in the derivations below. The *K* parameter characterizes the phase shift of the solution across the elementary cell of the periodic structure.

For certain frequency intervals, waves become evanescent. Such frequency intervals are referred to as the stop-bands.

The summary of the numerical method used here is given in appendix A.

### (a) Effective wave speed

**Figure 2.**
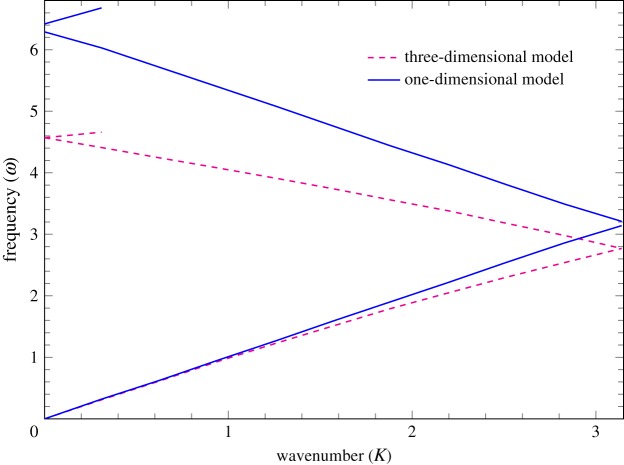
Dispersion curves for the unit cell without stent. The dashed line represents the dispersion curves obtained using the three-dimensional FEM model of [20], while the solid line represents the results of the one-dimensional model presented in this paper. (Online version in colour.)

The dispersion curves are plotted in the first Brillouin zone, specifically in the interval [0, *π*]. Figure 2 presents a comparison of the dispersion curves for an artery without stent (*C* = 1) corresponding to the one-dimensional model and the three-dimensional model of [20]. The actual frequency *v*_*d*_ (in Hz) in the three-dimensional model is related to the non-dimensional frequency *ω* according to

3.2 $$v_{d}=\frac{\omega c_{0}}{2\pi L}.$$

The reference pulse wave speed *c*_0_ is obtained from the dispersion diagram of the three-dimensional model of [20] for an unstented artery. Specifically, *c*_0_ is the group velocity *c*_0_ = *ω*/*K* calculated near *K* = 0.

As shown in figure 2, the one-dimensional model provides an upper bound (see the region in the neighbourhoods of *K* = *π* or *K* = 0) for the stop-band frequencies. This applies both to the first and second stop-bands. The three-dimensional computational model of [20] includes general vibration modes and the actual geometry of the stent. Here we use three-dimensional computation for calibration of the semi-analytical one-dimensional model, which is highly effective in the analysis of transmission–reflection of waves across the stented region. It is also noted that the one-dimensional model reflects correctly important features, including, for example, the growth in the magnitude of the group velocity along the acoustic band. The mean value of the normalized wave speed in the stented artery is evaluated using equation (2.14). We also note that the corresponding dimensional considerations for the actual blood vessels with the actual stents were included in the earlier paper [20].

**Figure 3.**
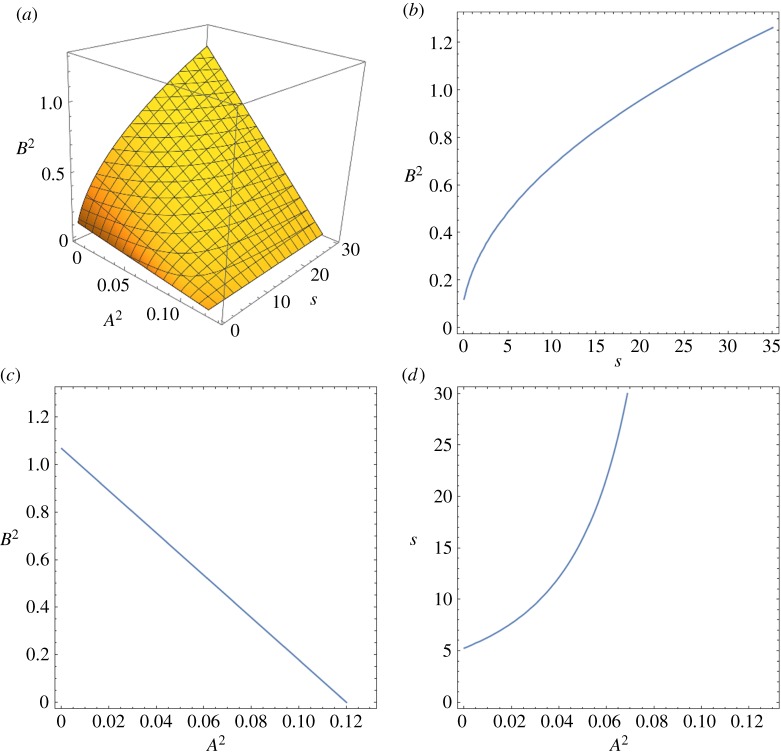
The effect of the parameters *A*^2^, *B*^2^, and *s* in (2.14) on the mean non-dimensional speed for a stented unit cell. Here we take *C* = 1.12. (*a*) Implicit solution of equation (2.14) for *A*^2^∈[0, 0.13], *B*^2^∈ [0, 1.3], *s*∈[0, 30]. (*b*) Contour plot for *A*^2^ = 0. (*c*) Contour plot for *s* = 25. (*d*) contour plot for *B*^2^ = 0.5. (Online version in colour.)

The parameters *A*^2^, *B*^2^ and *s* are calibrated such that the group velocity near the origin for the one-dimensional model matches the group velocity of the axisymmetric mode in the three-dimensional model of [20]. For the types of stents considered in [20], the mean non-dimensional speed in each unit cell is *C*≃1.12. In figure 3*a*, we plot the surface (2.14) for this value of *C* for *A*^2^∈[0, 0.13], *B*^2^∈[0, 1.3], *s*∈[0, 30]. Figure 3*b*–*d* describes the behaviour of the three parameters used in the definition of the mean non-dimensional velocity. For fixed *A*^2^, it can be noted that *B*^2^ increases with *s* (figure 3*b*). For fixed *s*, if *B*^2^ increases then *A*^2^ decreases (figure 3*c*). Finally, for fixed *B*^2^, *s* increases with *A*^2^ (figure 3*d*).

### (b) Stop-bands for waves in structured arteries

Here we consider the case of *A*^2^ = 0, which corresponds to an artery without a plaque.

**Figure 4.**
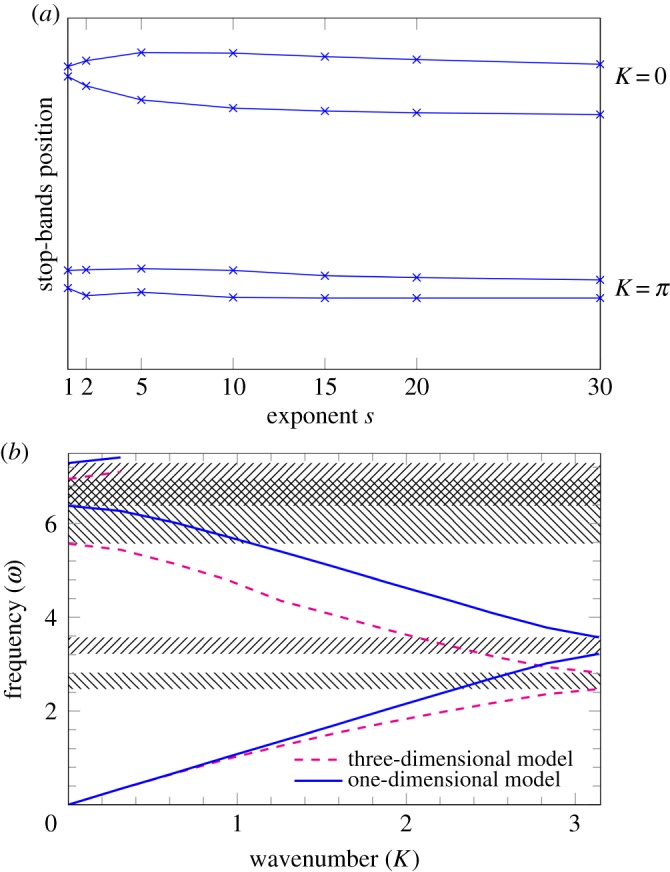
The first two stop-bands for the one-dimensional model discussed in §2 and the three-dimensional model of the pulsating wave. Part (*a*) shows the boundaries of the first two stop-bands (for *K* = 0 and *K* = *π*) as function of *s* for the one-dimensional model. Here *A*^2^ = 0, and *f*(*ξ*) = sin^2*s*^(*πξ*). *B*^2^ varies with *s* according to figure 3*b* (see also (2.11)). Part (*b*) illustrates the dispersion curves for *A*^2^ = 0, *B*^2^ = 0.97, *f*(*ξ*) = sin^40^(*πξ*). The dashed line represents the three-dimensional model (as in [20]), while the solid line corresponds to the one-dimensional model. (Online version in colour.)

The choices *s* = 1, *s* = 2, *s* = 5, *s* = 10, *s* = 15, *s* = 20 and *s* = 30 are substituted into equation (2.11) to obtain the position and width of the stop-bands for the dispersion curves at *K* = 0 and *K* = *π* for the stented case. The results are illustrated in figure 4*a*.

The width of the stop-band is considered to be an important parameter when comparing the one-dimensional approximation with the full three-dimensional model of [20].

A better approximation is to be expected for the first stop-band at *K* = *π*, since it is in the low-frequency regime. It is noted that, in general, higher values of *s* produce better results, with the best result in figure 4*a* being for *s* = 20, *B*^2^ = 0.97 (shown in figure 4*b*). There is extremely good correspondence at *K* = *π* between the stop-band widths, with 0.36 for both the one-dimensional (solid line) and three-dimensional (dashed line) models. For the higher value of *s* = 30, there again appears to be a good correspondence between the stop-bands, with the one-dimensional model predicting a somewhat smaller band-width compared to the three-dimensional model.

Interestingly, the extreme case of small parameter values (*s* = 1, *A*^2^ = 0, *B*^2^ = 0.24) in figure 4*a* results in a narrower stop-band for the one-dimensional model. The overlapping of the second stop-bands (illustrated by the cross-hatched areas in figure 4*b*) does not occur for *s* = 1. Results for *s* = 1 were also discussed in [1].

The new model proposed here allows for higher values of *s* to be incorporated into the analysis showing a significant improvement in agreement for sufficiently large *s*.

## 4. Transmission problem

In this section, the transmission problem for a stented blood vessel is considered. The volumetric flow rates in the reflection region, stented region and transmission region are assumed to be of the form

4.1a $$\begin{array}{rl} & Q_{R}=q_{R}(\xi )\exp\left(-i\omega \eta \right),\;-\infty <\xi <0, \end{array}$$

4.1b $$\begin{array}{rl} & Q=y(\xi )\exp\left(-i\omega \eta \right),\;0<\xi <n \end{array}$$

4.1c $$\begin{array}{rl} \mathrm{and}\;\; & Q_{T}=q_{T}(\xi )\exp\left(-i\omega \eta \right),\;n<\xi <\infty , \end{array}$$

respectively, where *ω* is the angular frequency.

**Figure 5.**
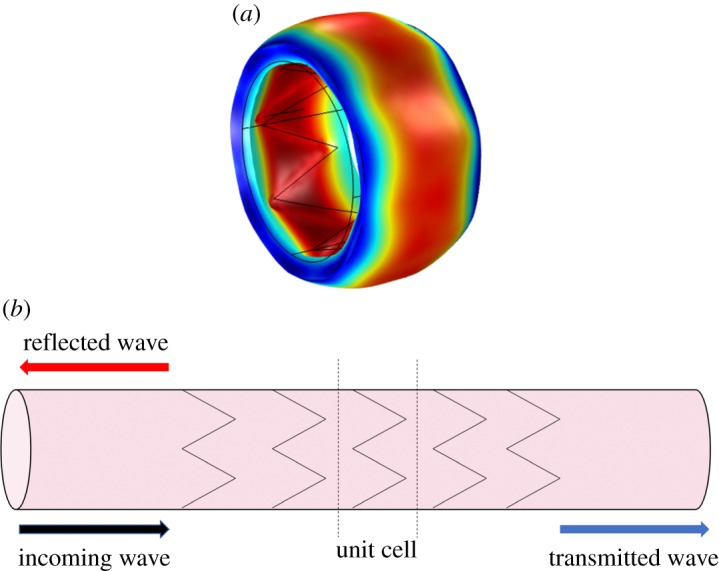
(*a*) The axisymmetric mode obtained using the three-dimensional model in [20]. (*b*) Reflected and transmitted waves in a stented artery. (Online version in colour.)

A diagram for the reflection–transmission problem is shown in figure 5*b*. The flow rate in the reflection region consists of the original incoming wave, with unit amplitude and the reflected wave which has amplitude *R*,

4.2 $$q_{R}(\xi )=\exp\left(i\omega \xi \right)+R\exp\left(-i\omega \xi \right).$$

In the transmission region, the outgoing wave is of amplitude *T*,

4.3 $$q_{T}(\xi )=T\exp\left(i\omega \xi \right).$$

The four interface conditions are

4.4a $$\begin{array}{rl} & q_{R}(0)=y(0)\;\mathrm{and}\;{\frac{dq_{R}}{d\xi }|}_{\xi =0}=Y(0), \end{array}$$

4.4b $$\begin{array}{rl} & q_{T}(n)=y(n)\;\mathrm{and}\;{\frac{dq_{T}}{d\xi }|}_{\xi =n}=Y(n), \end{array}$$

where *y*(*ξ*) and *Y* (*ξ*) are defined in (2.11) and (2.12). Equation (2.11) is solved numerically using the Galerkin method with finite-element discretization together with the interface conditions (4d).

### (a) Reduction to the Mathieu equation for the case *s* = 1

When *s* is chosen to be 1, the mean non-dimensional speed in each cell is given by

4.5 $$C=1+A^{2}+\frac{B^{2}}{2},$$

for both functions *f*(*ξ*) = sin^2^(*πξ*) and *f*(*ξ*) = cos^2^(*πξ*). Furthermore, using the formulae 2sin^2^(*ξ*) = 1 − cos(2*ξ*) and 2cos^2^(*ξ*) = 1 + cos(2*ξ*), and calling

4.6 $$\epsilon =\frac{B^{2}}{2C}=\frac{B^{2}}{2+2A^{2}+B^{2}}<1,$$

leads to the equation

4.7 $$\frac{d^{2}Y}{d\xi^{2}}+\frac{\omega^{2}}{C^{2}{\left(1\mp \epsilon \cos\left(2\pi \xi \right)\right)}^{2}}Y=0,$$

where the minus sign corresponds to the choice *f*(*ξ*) = sin^2^(*πξ*) and the plus sign to *f*(*ξ*) = cos^2^(*πξ*). Assuming now *ϵ*≪1 and noticing that |cos(2*πξ*)|≤1, the expansion

4.8 $${\left(1\mp \epsilon \cos(2\pi \xi )\right)}^{-2}=1\pm 2\epsilon \cos(2\pi \xi )+O(\epsilon^{2})\approx 1\pm 2\epsilon \cos(2\pi \xi ),$$

can be used. Substituting (4.8) into (4.7) and using the change of variable *πξ* = *z* we obtain the following standard Mathieu equation

4.9 $$\frac{d^{2}Y}{dz^{2}}+\left[a\pm 2q\cos(2z)\right]Y=0,$$

with

4.10a $$a={\left(\frac{\omega }{\pi C}\right)}^{2}$$

and

4.10b $$q=\epsilon {\left(\frac{\omega }{\pi C}\right)}^{2}=\epsilon a.$$

The general solution for the Mathieu equation [24] is given by

4.11 $$Y(\xi )=C_{1}M_{S}\left(a,-q,\pi \xi \right)+C_{2}M_{C}\left(a,-q,\pi \xi \right),$$

for *f*(*ξ*) = sin^2^(*πξ*), and

4.12 $$Y(\xi )=C_{1}M_{S}\left(a,q,\pi \xi \right)+C_{2}M_{C}\left(a,q,\pi \xi \right),$$

for *f*(*ξ*) = cos^2^(*πξ*), where *M*_*S*_ and *M*_*C*_ are the Mathieu functions, linearly independent solutions of (4.9) with the property that *M*_*S*_(*a*, *q*, 0) = 0 and *M*′_*C*_(*a*, *q*, 0) = 0. In what follows we consider the case *f*(*ξ*) = sin^2^(*πξ*) that corresponds to a periodic cell which is more compliant at its edges. Applying the Bloch–Floquet conditions (3a) to the Mathieu equation the following dispersion relation is obtained

4.13 $$\begin{array}{rl} & \left[M_{C}\left(a,-q,\pi \right)M{{\;}^{\prime }}_{S}\left(a,-q,0\right)+M_{C}\left(a,-q,0\right)M{{\;}^{\prime }}_{S}\left(a,-q,\pi \right)\right]\exp\left(iK\right)\\ & \;\;+M_{S}\left(a,-q,\pi \right)M{{\;}^{\prime }}_{C}\left(a,-q,\pi \right)=M{{\;}^{\prime }}_{S}\left(a,-q,\pi \right)M_{C}\left(a,-q,\pi \right)\\ & \;\;+M_{C}\left(a,-q,0\right)M{{\;}^{\prime }}_{S}\left(a,-q,0\right)\exp\left(2iK\right). \end{array}$$

The choice of parameters *A*^2^ and *B*^2^ is determined by the effective wave speed, as explained in §3a.

**Figure 6.**
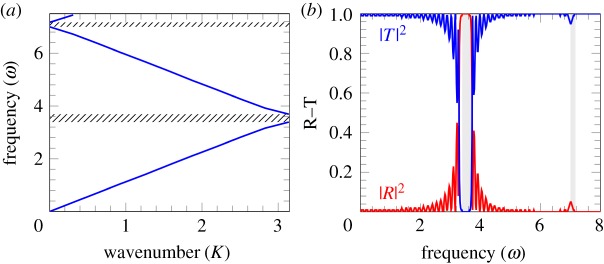
(*a*) The dispersion curves for the Mathieu equation. (*b*) The corresponding transmission problem for the Mathieu equation with *n* = 24 cells. The parameters defining the velocity are chosen to be *A*^2^ = 0, *B*^2^ = 0.24, *f*(*ξ*) = sin^2^(*πξ*), so that *C* = 1.12. (Online version in colour.)

As expected, the reflection at frequencies corresponding to the stop band is high, as illustrated in figure 6*b*, which shows the reflection and transmission coefficients |*R*|^2^ and |*T*|^2^ versus the frequency of the incident wave. We also note a relatively high reflection within the first pass-band region, especially in the neighbourhood of the stop-band boundary. Such reflection can be altered by changing the rate of localization within the structured stent. It can be noted that in figure 6*b* the second stop-band is not visible as it requires more cells to be considered.

### (b) Transmission for the higher-order sparse stent structure

**Figure 7.**
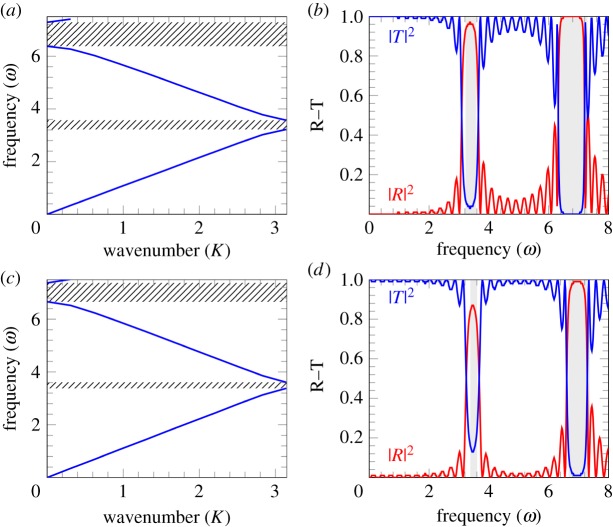
Dispersion curves (*a*,*c*) and corresponding reflection–transmission diagrams (*b*,*d*) for a stented artery, with *f*(*ξ*) = sin^40^(*πξ*) and *A*^2^, *B*^2^ defined from (2.14) for *C* = 1.12. (*a*) Dispersion curves for *A*^2^ = 0, *B*^2^ = 0.97. (*c*) Dispersion curves for *A*^2^ = 0.05, *B*^2^ = 0.58. (*b*) Transmission–reflection diagram for *A*^2^ = 0, *B*^2^ = 0.97, *n* = 12. (*d*) Transmission–reflection diagram for *A*^2^ = 0.05, *B*^2^ = 0.58, *n* = 12. (Online version in colour.)

At higher values of the exponent *s* characterizing the sparse stent structure, the transmission diagrams are shown in figure 7*b*,*d*. The choice of parameters is the same as in the dispersion diagrams of figure 7*a*,*c*.

In figure 7*b*, the shaded areas match the hatched areas in figure 7*a*, as expected. Accordingly, the stop-bands correspond to the high reflection of the incident wave.

The reflection at frequencies corresponding to the first pass-band appears to be smaller for highly localized structured stents compared with the case of *s* = 1. Also, the secondary stop-band in diagrams (*a*) and (*c*) of figure 7 corresponds to a higher reflection, if the frequency of the incident wave includes higher-order harmonics; this is likely to be the case in the transition regimes or abnormalities leading to an irregular heart beat.

Transmission diagrams for the case of *s* = 20 are shown in figure 7*b*,*d* for the value of *C* = 1.12; they correspond to different values of *A*^2^ and *B*^2^ evaluated using (2.14), which represent an alteration in the stiffness of the artery and of the stent. This is shown to have an effect on reflection in the second pass-band, while the reflection in the first pass-band remains low.

## 5. Dispersion curves and transmission problem for overlapping stents

Stent overlapping is a common procedure in vascular surgery, especially in the femoral artery where two or three overlapping stents may be needed to cover the whole area affected by stenosis [25–29]. However, this procedure is often associated with increased risk in the clinical outcome.

**Figure 8.**
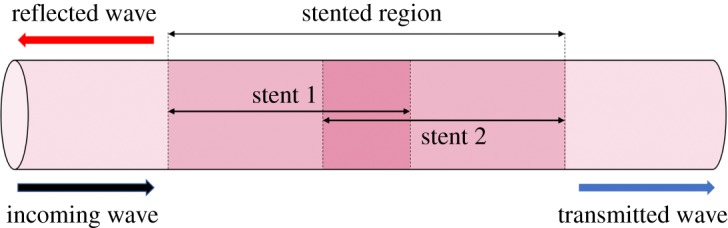
Scheme representing the case of an artery with two stents that overlap. (Online version in colour.)

In this section, the Bloch–Floquet analysis and the transmission problem for a stented artery with overlapping stents will be discussed. The problem is represented in figure 8. The non-dimensional velocity in the artery is assumed to be

5.1 $$\frac{c(\xi )}{c_{0}}=\left\{ \begin{array}{ll} 1, & \xi \in (-\infty ,0)\\ 1+A^{2}+B^{2}\sin^{2s}(\pi \xi )\;\mathrm{or}\;1+A^{2}+B^{2}\cos^{2s}(\pi \xi ), & \xi \in (0,n)\\ \sqrt{2}(1+A^{2}+B^{2}\sin^{2s}(\pi \xi ))\;\mathrm{or}\;\sqrt{2}(1+A^{2}+B^{2}\cos^{2s}(\pi \xi )), & \xi \in (n,m)\\ 1+A^{2}+B^{2}\sin^{2s}(\pi \xi )\;\mathrm{or}\;1+A^{2}+B^{2}\cos^{2s}(\pi \xi ), & \xi \in (m,d)\\ 1, & \xi \in (d,+\infty ), \end{array}\right.$$

where *n* is the number of stent coils constituting the first stent excluding the overlapping region, *m* is the number of stent coils in the overlapping region, and *d* is the number of stent coils constituting the second stent excluding the overlapping region.

Assuming that the solution of equation (2.9) is of the form *Q*_*i*_ = *y*_*i*_(*ξ*) e^−i*ωη*^, where *ω* is the angular frequency and *i* = 1, 2, 3, the functions *y*_*i*_(*ξ*) then satisfy the equation

5.2 $$\frac{d}{d\xi }\left({\left[\lambda (\xi )(1+A^{2}+B^{2}f(\xi ))\right]}^{2}\frac{dy_{i}}{d\xi }\right)+\omega^{2}y_{i}=0,$$

*λ*(*ξ*) is the piecewise constant function, which is equal to 1 in the stented region without overlapping, and it is equal to $\sqrt{2}$ in the overlapping region. We also introduce the following notation:

5.3a $$\begin{array}{rl} & Y_{1}={\left[1+A^{2}+B^{2}f(\xi )\right]}^{2}\frac{dy_{1}}{d\xi }, \end{array}$$

5.3b $$\begin{array}{rl} & Y_{2}=\sqrt{2}{\left[1+A^{2}+B^{2}f(\xi )\right]}^{2}\frac{dy_{2}}{d\xi } \end{array}$$

5.3c $$\begin{array}{rl} \mathrm{and}\;\; & Y_{3}={\left[1+A^{2}+B^{2}f(\xi )\right]}^{2}\frac{dy_{3}}{d\xi }. \end{array}$$

### (a) Stop-bands and reflected energy

The Bloch–Floquet analysis and the transmission problem for a stented artery with an overlapping region is discussed in this section. The total length of the unit periodic cell for the stented artery with an overlapping region is given by *N* = *n* + *m* + *d*. Equation (5.2) is solved numerically using the Galerkin method together with the following Bloch–Floquet conditions applied at the end parts of the unit cell *ξ* = 0 and *ξ* = *N*:

5.4a y(N)=y(0)exp⁡(iKN)

and

5.4b $$y_{\xi }(N)=y_{\xi }(0)\exp\left(iKN\right),$$

where *K*, as before, is the one-dimensional Bloch–Floquet parameter. Continuity of the solution between the different regions of the stented artery is also imposed.

For the transmission problem, the volumetric flow rate in the reflected region, stented regions, overlapping region and transmission region are of the form

5.5a $$\begin{array}{rl} & Q_{R}=q_{R}(\xi )\exp\left(-i\omega \eta \right),\;-\infty <\xi <0, \end{array}$$

5.5b $$\begin{array}{rl} & Q_{1}=y_{1}(\xi )\exp\left(-i\omega \eta \right),\;0<\xi <n, \end{array}$$

5.5c $$\begin{array}{rl} & Q_{2}=y_{2}(\xi )\exp\left(-i\omega \eta \right),\;n<\xi <n+m, \end{array}$$

5.5d $$\begin{array}{rl} & Q_{3}=y_{3}(\xi )\exp\left(-i\omega \eta \right),\;n+m<\xi <n+m+d \end{array}$$

5.5e $$\begin{array}{rl} \mathrm{and}\;\; & Q_{T}=q_{T}(\xi )\exp\left(-i\omega \eta \right),\;n+m+d<\xi <\infty . \end{array}$$

The flow rate *q*_*R*_ in the reflected region and *q*_*T*_ in the transmitted region are defined in the same way as the case of a stented artery without overlapping (see (4.2) and (4.3)). Recalling (5.2), in the stented region the following equations are valid:

5.6 $$y_{i}=-\frac{1}{\omega^{2}}\frac{dY_{i}}{d\xi },\;i=1,2,3.$$

Consequently, the eight interface conditions are

5.7a $$\begin{array}{rl} q_{R}(0) & \;=y_{1}(0)\;\mathrm{and}\;{\frac{dq_{R}}{d\xi }|}_{\xi =0}=Y_{1}(0), \end{array}$$

5.7b $$\begin{array}{rl} y_{2}(n) & \;=y_{1}(n)\;\mathrm{and}\;{\frac{dy_{2}}{d\xi }|}_{\xi =n}=Y_{1}(n), \end{array}$$

5.7c $$\begin{array}{rl} y_{2}(n+m) & \;=y_{3}(n+m)\;\mathrm{and}\;{\frac{dy_{2}}{d\xi }|}_{\xi =n+m}=Y_{3}(n+m), \end{array}$$

5.7d $$\begin{array}{rl} q_{T}(n+m+d) & \;=y_{3}(n+m+d)\;\mathrm{and}\;{\frac{dq_{T}}{d\xi }|}_{\xi =n+m+d}=Y_{3}(n+m+d). \end{array}$$

**Figure 9.**
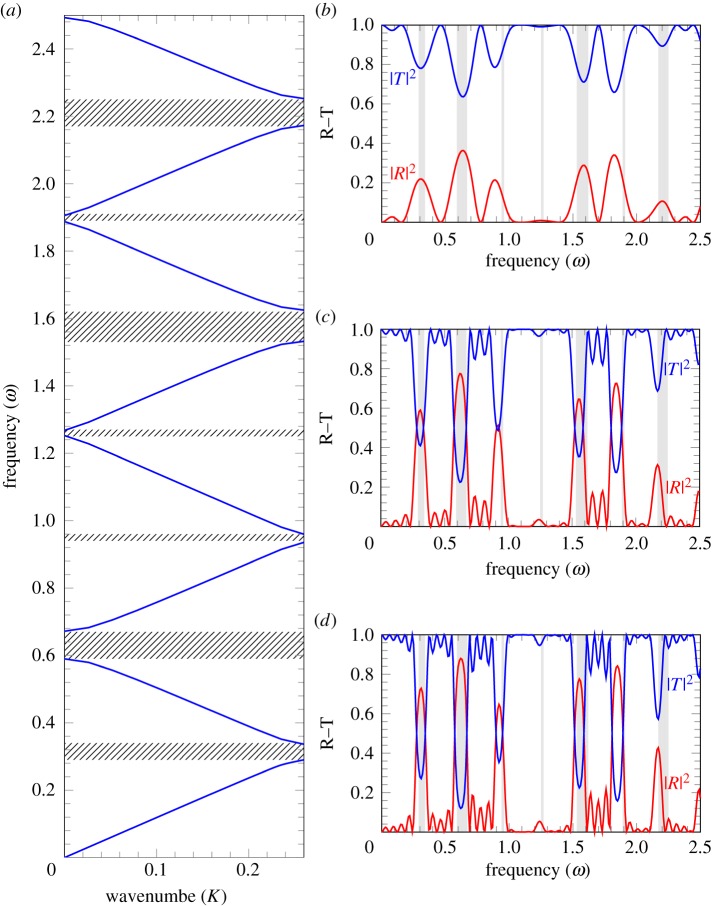
Dispersion curves (*a*) and reflection–transmission diagrams (*b*–*d*) for the problem of overlapping stents. The parameters for the mean non-dimensional speed are chosen to be *A*^2^ = 0, *B*^2^ = 0.97, *f*(*ξ*) = sin^40^(*πξ*), *m* = *n* = *d* = 4. (*a*) Dispersion curves. The dashed area represents the stop-band regions. (*b*) Reflection–transmission diagram with two periodic cells. (*c*) Reflection–transmission diagram with four periodic cells. (*d*) Reflection–transmission diagram with five periodic cells. (*b*–*d*) The shaded areas correspond to the stop-band regions. (Online version in colour.)

**Figure 10.**
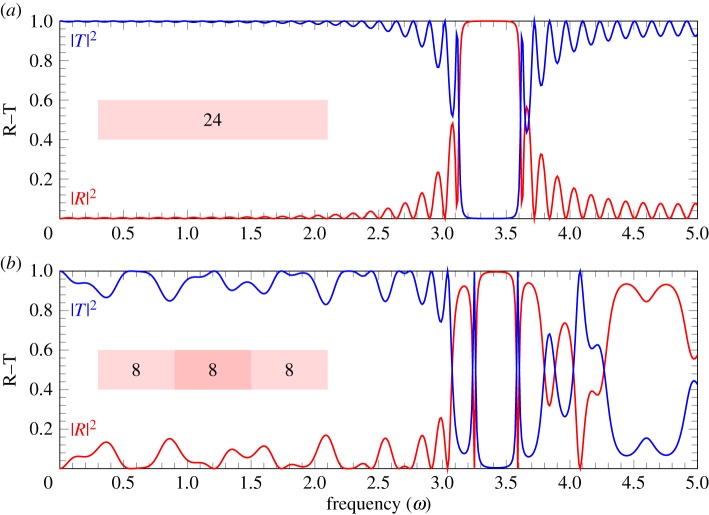
Reflection–transmission diagrams for (*a*) long stent composed of 24 periodic cells with no overlapping region (*n* = 24, *m* = *d* = 0); (*b*) two stents composed of 16 periodic cells each with an overlapping region of 8 cells (*n* = *m* = *d* = 8). The parameters for the mean non-dimensional speed used in the calculations are *A*^2^ = 0, *B*^2^ = 0.97, *f*(*ξ*) = sin^40^(*πξ*). (Online version in colour.)

The dispersion curves resulting from the Bloch–Floquet analysis are plotted in figure 9*a*. The analysis is representative for the case of two stents composed of eight coils each, and an overlapping area composed of four coils. Observing the dispersion curves in figure 9*a*, it is possible to note that the range of frequencies in the low-frequency regime corresponding to the pass-band in figure 4*b* (that is where the wave propagates), now contains a number of stop-bands (where the wave does not propagate). Figure 9*b*,*d* represents the reflection–transmission diagrams for a different number of periodic cells. It can be noted that as more periodic cells are considered (that is stents with multiple overlapping regions), the reflection in the range of frequencies corresponding to the stop-bands becomes higher. Figure 10 shows the comparison between a long stent composed of 24 periodic cells and two stents composed of 16 periodic cells each with an overlapping region composed of eight periodic cells. It can be observed that in the low-frequency regime, the energy reflected is higher in the case when the stents do overlap, which may increase the risk of restenosis if overlapping stents are used.

## 6. Conclusion

The paper brings a new insight into the analysis of a dynamic response of multi-scale stented vascular systems, which are often neglected. It is a common knowledge in medical acoustics that branching blood vessels reflect waves, and surgeons also observe that vascular blockages and aneurysms often occur near vascular junctions.

Not surprisingly, stents themselves may act as wave scatterers and reflect waves, especially in the transitional regimes when the rate of pulsation changes rapidly. The practical consequences are in secondary vascular blockages and formation of standing waves in the blood vessels.

In particular, the connection between dispersion properties and transmission–reflection of waves for different values of the stenting parameters has been analysed in detail here, which has provided a valuable material for assessment of performance of stents in different transitional regimes. This also includes overlapping stents, where elastic stiffness increases in the regions of overlap; it has been demonstrated that such configurations may lead to additional wave reflection and formation of stop-bands in the dispersion diagram.

The simplicity of the model, presented in this paper, makes it appealing to both researchers and medical practitioners, as it enables one to obtain ballpark ranges for values of stenting parameters that may lead to formation of high wave reflection in the transitional regimes.

## Data accessibility

This article has no experimental data. All computational results are reproducible.

## Appendix A. Summary of the numerical approach

The variational form of the governing equation (2.11) for the stented region is to find *y*∈*V* ⊂*H*^1^(0, *n*) and R,T∈C (reflection–transmission problem) or K∈R (Bloch–Floquet analysis) such that

A 1 $${\left[{\left(1+A^{2}+B^{2}f(\xi )\right)}^{2}wy_{\xi }\right]}_{0}^{n}-\int_{0}^{n}{\left(1+A^{2}+B^{2}f(\xi )\right)}^{2}wy_{\xi }\;d\xi +\omega^{2}\int_{0}^{n}wy\;d\xi ,$$

for all suitable weight functions *w*⊂*H*^1^(0, *n*) and ω∈R, where the standard notation *H*^1^(0, *n*)≡*W*^1,2^(0, *n*) for Sobolev (Hilbert) spaces is adopted.

In particular, for the reflection–transmission problem it is

A 2 $$V=V_{RT}=\{u\in H^{1}(0,n):u(0)=1+R,\;u(n)=T\;e^{i\omega n}\},$$

with the value for *y*_*ξ*_ at *ξ* = 0 and *ξ* = *n* defined in (4.4a) and (4.4b), respectively.

For the Bloch–Floquet analysis, it is

A 3 $$V=V_{\mathrm{BF}}=\{u\in H^{1}(0,n):n=1,\;u(n)=u(0)\;e^{iK}\},$$

with *y*_*ξ*_(1) = *y*_*ξ*_(0) e^i*K*^.

Piecewise Lagrange interpolation of order *k*≥1 is introduced for the finite-element discretization of the above variational problem with a total of *N* elements. The discrete solution space is

A 4 $$V^{h}=\{u_{h}\in V:u_{h}{|}_{I_{i}}\in P_{k}(I_{i}),\;1\leq i\leq N\},$$

where *u*_*h*_|_*I*_*i*__ denotes the restriction of *u*_*i*_ in element *I*_*i*_ and *P*_*k*_ denotes the set of polynomials of degree at most *k*. For piecewise linear interpolation, it is *u*_*h*_|_*I*_*i*__∈*P*_1_(*I*_*i*_) and the standard Bubnov–Galerkin method with a uniform mesh size *h* = *n*/*N* can be employed using the linear shape functions *N*^(*i*)^,  *i* = 1, 2 and Gauss quadrature for the ‘stiffness’ integrals

A 5 $$\int_{I_{i}}{\left(1+A^{2}+B^{2}f(\xi )\right)}^{2}N_{\xi }^{\left(i\right)}N_{\xi }^{\left(j\right)}\;d\xi ,\;1\leq i,j\leq 2.$$

The following stiffness and mass type finite-element matrices can be used effectively for *h* → 0

A 6 $$-\frac{{\left(1+A^{2}+B^{2}f(m_{i})\right)}^{2}}{h}\left[\begin{array}{cc} 1 & -1\\ -1 & 1 \end{array}\right],\;\frac{\omega^{2}h}{6}\left[\begin{array}{cc} 2 & 1\\ 1 & 2 \end{array}\right],$$

where *m*_*i*_ is the midpoint of *I*_*i*_. For higher frequencies, increase of the polynomial degree along with increasing the number of elements can be used.

Both the reflection–transmission and Bloch–Floquet problems can be numerically solved by using the finite-element matrices in (A 6), the definitions of the solution spaces (A 2) and (A 3) and the boundary data for d*y*/d*ξ*, for different values of the frequency. Standard connectivity arrays and FEM assembly procedures apply ([21]). The connectivity array in this problem is the same as that for a one-dimensional bar/rod element. We note that as the frequency increases the discretization needs to become finer. In our examples, very fine meshes were used in all cases, with further mesh size reduction for validation of convergence. The standard method of Lagrange multipliers is used to enforce Dirichlet or Periodic data at the boundary.
